# TIM-1 and Tiny-TIM as Robust In Vitro Models for Oral Biopharmaceutics: Evidence from an International Ring Study

**DOI:** 10.3390/pharmaceutics18040400

**Published:** 2026-03-24

**Authors:** Connor O’Farrell, Robert Havenaar, Mark McAllister, Bart Hens, Richard Barker, Álvaro López Mármol, Andrea Ansari, Tom Ooms, Ronald Schilderink, Robert Schwabe, James Butler, Malgorzata Stróžyk, Tânia Martins Garcia, Dyko Minekus, Inese Sarcevica, Kieran Smith, Irena Tomaszewska, Eleanor Jones, Hannah Batchelor, Susann Bellmann

**Affiliations:** 1 InnoGI Technologies, Thijsseweg 11, 2629 JA Delft, The Netherlands; tania.garcia@innogitechnologies.com (T.M.G.); dykomin@gmail.com (D.M.); susann.bellmann@nizo.com (S.B.); 2 Netherlands Organisation for Applied Scientific Research (TNO), Utrechtseweg 48, 3704 HE Zeist, The Netherlands; rbhvnr.zeist@gmail.com (R.H.);; 3 Drug Product Design, Pfizer, Discovery Park, Ramsgate Road, Sandwich CT13 9ND, UK; mark.mcallister@biowaived.com (M.M.); bart.hens@pfizer.com (B.H.); inese.sarcevica@biowaived.com (I.S.); kieran.smith@biowaived.com (K.S.); irena.tomaszewska@merck.com (I.T.); 4 Global Product Development, Pharmaceutical Technology & Development, Operations, AstraZeneca, Macclesfield SK10 2NA, UK; richard.barker2@astrazeneca.com; 5 Synthetic Molecules CMC R&D, AbbVie Deutschland GmbH & Co. KG, Knollstrasse, 67061 Ludwigshafen, Germany; alvaro.lopezmarmol@abbvie.com (Á.L.M.); andrea.wahl@abbvie.com (A.A.); 6 Johnson & Johnson, Turnhoutseweg 30, 2340 Beerse, Belgium; 7 Material and Analytical Sciences, Research and Development, Boehringer Ingelheim Pharmaceuticals, Inc., Ridgefield, CT 06877, USA; robert.schwabe@boehringer-ingelheim.com; 8 GlaxoSmithKline R&D, Harris’s Lane, Ware SG12 0DX, UK; james.m.butler@gsk.com; 9 PozLab Sp. z o.o. Grupa Selvita, 6 Kobaltowa Str., 62-002 Zlotniki, Poland; malgorzata.strozyk@selvita.com; 10 Strathclyde Institute of Biopharmaceutical Sciences, University of Strathclyde, Glasgow G1 1XQ, UK; eleanor.jones@strath.ac.uk (E.J.); hannah.batchelor@strath.ac.uk (H.B.)

**Keywords:** TIM-1, tiny-TIM, in vitro gastrointestinal model, in vitro dissolution, biorelevant dissolution, bioaccessibility

## Abstract

**Background/Objectives:** Biorelevant in vitro dissolution testing is used increasingly to predict complex mechanisms in the gastrointestinal (GI) tract that determine oral bioavailability. However, the limited use of non-compendial systems is driven by the lack of widely accepted, standardized validation frameworks. This ongoing gap continues to restrict their adoption relative to United States Pharmacopeia (USP) apparatus. While the physiological relevance and biopredictive capabilities of the tiny-TIM and TIM-1 in vitro GI models have been demonstrated in previous studies, their inter-laboratory reproducibility has not been systematically established. Therefore, this international ring study evaluates the reproducibility of in vitro simulations of GI transit and absorption of paracetamol in fasted- and fed-state conditions in tiny-TIM and TIM-1. **Methods**: Three laboratories used TIM-1 and five used tiny-TIM to simulate oral administration of a 500 mg paracetamol solution to a healthy adult. Paracetamol solution was selected as a well-characterized and widely available BCS I compound to minimize formulation and solubility effects and focus on system performance, enabling the generation of a generic validation dataset for the reproducibility of TIM experiments. **Results**: Paracetamol bioaccessibility profiles were repeatable and reproducible (all pairwise f_2_ > 50). Maximum differences in total bioaccessible paracetamol were 0.9% (TIM-1) and 2.8% (tiny-TIM) within laboratories and 3.4 and 5.9% between laboratories. Inter-lab variability at individual time points remained <4.0% (fasted) and 5.2% (fed). Both TIM models produced biopredictive metrics, correctly predicting no food effect on total paracetamol bioaccessibility and capturing delayed t_max_. Gastric and intestinal environments showed repeatable pH, temperature, and GI transit characteristics, with fluctuations across transit stages that mirrored reported in vivo patterns. **Conclusions**: These results demonstrate that TIM systems can reproducibly simulate gastrointestinal conditions across laboratories and generate consistent measurements of drug product performance, despite the complexity of the dynamic processes involved. While this evaluation involving a single BCS I drug solution should not be directly extrapolated to experiments with poorly soluble compounds or different formulations, it supports the use of TIM systems as robust in vitro models in drug product development. This study provides a standardized, inter-laboratory, baseline performance dataset to support regulatory submissions incorporating TIM data and enable more confident interpretation of TIM experiments.

## 1. Introduction

To successfully predict the in vivo exposure of oral drug products, in vitro models must provide reliable and well-understood predictions of release, dissolution and absorption of the active pharmaceutical ingredient (API). Achieving this requires close replication of the complex and dynamic intraluminal environment of the gastrointestinal (GI) tract. The European IMI project Oral Biopharmaceutics Tools (OrBiTo) evaluated predictive biopharmaceutical tools for oral drug delivery and reviewed a range of in vitro dissolution and GI simulation systems [1,2,3]. Since these reviews, biorelevant in vitro methods have been increasingly used to predict complex GI mechanisms controlling oral bioavailability, but compendial United States Pharmacopeia (USP) methods are still most frequently used in the literature [4]. These USP systems have value for routine high-throughput quality control (QC) dissolution testing at the commercial-scale to determine product quality and ensure batch-to-batch consistency. However, a lack of complexity and physiological relevance limits their biopredictivity and sensitivity in formulation development and in studying more complex mechanisms that occur during GI transit, such as supersaturation, precipitation, luminal degradation and bile salt/food-mediated solubility effects.

Among the in vitro GI models reviewed in the OrBiTo project are the tiny-TIM and TIM-1 models of the TIM Platform (InnoGI Technologies, Delft, The Netherlands, formerly The TIM Company), which simulate human GI transit, capturing luminal conditions in the stomach and small intestine [5]. The TIM systems have been described in detail elsewhere [6,7]. The original TIM-1 system was developed in the early 1990s by TNO (Zeist, The Netherlands) and consists of compartments representing the stomach, duodenum, jejunum and ileum [8]. The tiny-TIM was developed as a less labour-intensive version of TIM-1, with an advanced gastric compartment (TIM-AGC) in conjunction with a single intestinal compartment to simulate average small-intestinal conditions. 

While clinical and preclinical studies provide valuable pharmacokinetic (PK) results, they do not provide information on GI exposure or local GI concentrations that may affect the overall PK. TIM systems have been used to develop a mechanistic understanding of clinical observations and address regulatory questions [6,9], particularly unexpected results that arise due to formulation-related factors and interactions between the drug product and components of the dynamic GI luminal environment. As a result, TIM systems are increasingly used in pharmaceutical development, often in combination with physiologically based biopharmaceutics modelling (PBBM), for formulation development, candidate selection, clinical risk reduction and minimization of clinical bridging studies [10]. These integrated approaches enable mechanistic investigation under controlled and physiologically relevant conditions, thereby reducing reliance on animal studies, consistent with the 3Rs principles (Replacement, Reduction and Refinement). 

During a TIM experiment, absorption from the small-intestinal lumen is mimicked using a filtration system to remove intestinal fluids containing solubilized drug and digested food components. The mass of drug present in the filtrate is referred to as the bioaccessible fraction (in the absence of enterocyte permeation mechanisms, rather than the absorbed fraction) and can be used to estimate the in vivo absorption profile and fraction absorbed. TIM data have been integrated into model-informed drug development (MIDD) strategies by generating in vitro–in vivo correlations (IVIVCs) [11,12,13], establishing drug-product-specific PBBMs by directly using TIM data as a biopredictive input [14,15] and as a means of supportive verification for physiologically based pharmacokinetic (PBPK) predictions [16,17]. Combined TIM and PBPK approaches have also been applied to predict in vivo performance in cases where PBPK modelling alone failed to do so [14].

While the physiological relevance and biopredictive performance of the TIM systems have been demonstrated [3,6,15], systematic evaluation of their repeatability and reproducibility across laboratories remains limited and is needed to support broader regulatory confidence. This need extends to alternative biorelevant in vitro gastrointestinal models more generally, for which high-level regulatory guidance is currently absent, despite increasing support from initiatives such as the FDA Modernization Act 2.0 and broader adoption of New Approach Methodologies (NAMs). 

To address this gap, this international ring study assesses the intra-lab repeatability and inter-lab reproducibility of the tiny-TIM and TIM-1 systems under standardized fasted- and fed-state conditions. This evaluation includes both the reproducibility of bioaccessibility measurements and key intraluminal environmental parameters across multiple laboratories. Paracetamol solution was selected as a model BCS I formulation to minimize formulation-dependent variability and solubility effects and allowing more direct evaluation of system performance. The study is designed to generate a generic validation dataset for repeatability and inter-laboratory reproducibility to facilitate transparent interpretation of TIM experiments and to support regulatory interactions that include TIM data, as suggested by the TIM Expert Working Group [6].

## 2. Materials and Methods

### 2.1. Participating Laboratories

The experiments conducted with the tiny-TIM and TIM-1 were performed by seven different laboratories in six different countries, as summarized in Table 1. Operators in all laboratories received identical formal training. To minimize inter-operator variability, all participating laboratories followed harmonized standard operating procedures (SOPs) and working documents. These documents defined standardized protocols for TIM system initialization, experimental parameters, and preparation and administration of the dose and gastric intake, as well as the analytical methods used for sample analysis.

### 2.2. Test Products and Gastric Intake

Paracetamol (acetaminophen; Sigma A7085) was procured from Sigma Aldrich (Merck, Burlington, MA, USA) and distributed to all participating laboratories. A 500 mg dose of paracetamol was administered to the gastric compartment of the TIM systems as an oral solution. In the fasted state, this was dissolved in a dose volume of 270 mL of water to simulate a standard glass of water. In the fed state, the dose was dissolved in a volume of 285 mL (300 g), consisting of water (70 mL), gastric electrolytes (80 g) and a homogenized (to simulate mastication) high-fat meal (HFM, 150 g, ~135 mL). The HFM was prepared at laboratory 5 in one batch and distributed to all participating laboratories. The meal contained approximately 50% fat, 20% protein, and 30% carbohydrates for the total energy intake and was composed of eggs, bacon, toast, potatoes, milk, butter, and margarine as recommended by the FDA for clinical studies [18].

The gastric intake was adjusted to pH 3.0 ± 0.1 and 6.5 ± 0.1 in the fasted and fed state respectively, using hydrochloric acid. For the fed state, the homogenized HFM was heated to 37 °C and transferred into the gastric compartment.

### 2.3. In Vitro Gastrointestinal Models (Tiny-TIM and TIM-1)

**Figure 1:**
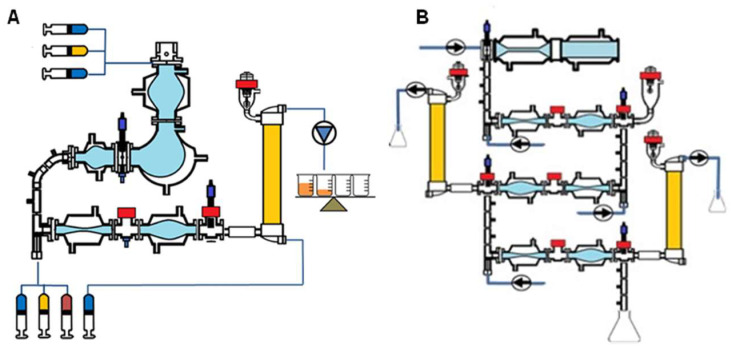
In vitro gastrointestinal models: Schematic diagrams of (**A**) the tiny-TIM [21]; (**B**) the TIM-1 [5].

Schematic diagrams of the tiny-TIM and the TIM-1 are shown in Figure 1A and 1B, respectively. TIM-1 is equipped with a horizontal stomach compartment. In contrast, the tiny-TIM is fitted with the TIM-AGC, which more closely replicates gastric architecture and motility [19,20,21]. While the TIM-AGC is by default part of the tiny-TIM, it can be implemented in TIM-1 as an alternative to the conventional horizontal stomach compartment for formulations that are more sensitive to gastric hydrodynamics (not implemented in this study).

### 2.4. Simulated Fasted- and Fed-State Conditions

Table 2 displays the control parameters for gastric emptying and pH profiles. The temperature, pH, and gastric pressure were continuously monitored (temporal resolution of 1 min) using in-line probes, and the volumes of the secreted and absorbed (filtrate) fluids were measured using load cells integrated into the TIM systems. These data were used to assess the performance of the control systems in maintaining repeatable and reproducible intraluminal conditions. The gastric emptying time is when a mimicked housekeeper wave (HKW) occurs, at which point the contents of the stomach are emptied into the small intestine of tiny-TIM or the duodenum of TIM-1 [21].

Each participating laboratory installed the same hollow fibre polysulfone filter with a pore size of 50 nm (discussed in detail in [6]) in their tiny-TIM and TIM-1 systems. The filtrate from the small-intestinal compartment of tiny-TIM and the jejunal and ileal compartments of TIM-1 was collected over defined time intervals (15, 30 or 60 min in tiny-TIM and 60 min intervals for TIM-1 in this study) over a duration of 300 min. The ileum effluent of TIM-1 was collected via the ileo-caecal valve (tiny-TIM was operated with no ileal effluent) following the same schedule. Two 1 mL subsamples of both the pooled filtrate and ileal effluent samples were each mixed with 4 mL acetonitrile/water (50/50, *v*/*v*) and stored at 2–10 °C. Sample analysis took place at each individual lab within 24 h.

The collected samples and control samples (blank TIM media spiked with paracetamol reference standard at three known concentrations) were analysed for paracetamol concentration using ultra-performance liquid chromatography (UPLC), with a Phenomenex Luna 3 µm C_18_ column at 40 °C and detection at 254 nm, or high-performance liquid chromatography (HPLC) using an equivalent method (laboratories 4 and 6). The mass of paracetamol in the filtrate in each time interval ($m_{filtrate}$) was calculated by multiplying the measured paracetamol concentration in the subsample ($C_{filtrate\;sample})$ by the volume (assuming fluid density = 1.0 g mL^−1^) of the total filtrate sample ($V_{filtrate})$, as in Equation (1).

(1) $$C_{filtrate\;sample}\times V_{filtrate}=m_{\;filtrate}$$

### 2.5. Total Bioaccessible and Recovered API

The bioaccessible fraction of paracetamol ($f_{av}$) was expressed as a percentage of dose (%D) as in Equation (2). Throughout this work, “bioaccessibility” refers to the fraction available for absorption in the intestinal lumen, not to be confounded with “bioavailability” which includes downstream PK processes beyond the scope of the TIM models.

(2) $$f_{av}\;(\%D)=\frac{m_{\;filtrate}}{dose}\times 100$$

At the end of each experiment, a mass balance was carried out to determine the recovery of paracetamol. The recovered mass ($m_{rec.}$) is defined as the sum of the bioaccessible mass ($m_{filtrate}$) and any paracetamol remaining in the residual luminal fluid and, for TIM-1, the ileal effluent at the end of the experiment. The $f_{av}$ was also expressed as a percentage for the recovered API, %R (Equation (3)).

(3) $$f_{av}\;(\%R)=\frac{m_{\;filtrate}}{m_{rec.}}\times 100$$

### 2.6. Cumulative Bioaccessibility Profile Analysis

Cumulative bioaccessibility profiles were calculated for each experiment and their similarity across replicates and between labs was assessed using the f_2_ similarity factor (Equation (4)), in accordance with regulatory guidance (e.g., FDA, EMA) for dissolution profile analysis. Firstly, f_2_ analysis of replicate profiles (the same TIM system, the same fasted or fed conditions and the same lab) was carried out to assess repeatability of the cumulative bioaccessibility profiles. Secondly, reproducibility was assessed by pairwise evaluation of the mean cumulative TIM bioaccessibility profile for each group. f_2_ ≥ 50 is generally accepted to indicate similar profiles.

(4) $$f_{2}=50\times \log\left({\left[1+\frac{1}{n}\sum_{t=1}^{n}{\left(A_{t}-B_{t}\right)}^{2}\right]}^{-\frac{1}{2}}\times 100\right)$$

A_t_ and B_t_ represent the values of labs A and B (for example) at each time point and n is the number of time points (excluding t = 0 min). Although regulatory guidance typically applies f_2_ to percent-dissolved profiles generated from minimum 12 dosage units under compendial dissolution conditions, the approach was applied here to cumulative TIM bioaccessibility profiles as a pragmatic measure of similarity for standardized dynamic GI simulations.

### 2.7. TIM Bioaccessibility Metrics

The TIM bioaccessibility metrics evaluated in this work were the fraction bioaccessible at 3 and 5 h (f_av,3_ and f_av,5_), the maximum fraction bioaccessible (f_av_,_max_) and t_max_, summarized in Table 3. 

For the calculation of f_av_,_max_, filtrate sampling intervals of different durations were normalized to the longest interval (30 min for tiny-TIM and 60 min for TIM-1, in this study) to enable direct comparison between f_av_,_max_ values across sampling intervals throughout the experiments. Where shorter sampling intervals were used, the bioaccessible paracetamol measured in those intervals was pooled to obtain intervals of equivalent duration. Longer intervals were not subdivided, as the rate of bioaccessibility within these intervals could not be resolved.

These metrics were first calculated per lab and presented as mean ± coefficient of variation (CV, %), where CV was calculated according to Equation (5), where σ is the standard deviation and $\bar{x}$ is the arithmetic mean of the replicate data. The CV was used to assess repeatability, while comparisons between labs were used to assess reproducibility of these metrics.

(5) $$CV=100*\;\;\frac{\sigma }{\bar{x}}$$

Data from all labs (on an experiment-replicate level) were then sorted into 4 groups, according to system type (tiny-TIM or TIM-1) and simulated nutritional state (fasted or fed). Within each group the geometric mean (GM) and 90% confidence interval (CI) were evaluated (Appendix A). Food effect was assessed by evaluating equivalence of fasted vs. fed conditions within tiny-TIM and TIM-1. Consistent with EMA [22] and FDA [23] bioequivalence guidelines, geometric mean ratios (GMRs) were calculated between groups, which were considered equivalent if both the point estimate and its associated 90% CI fell within the range of 80–125%. A full statistical breakdown of the methods can be found in Appendix A.

**Table 3:** TIM bioaccessibility metrics overview.

TIM Bioaccessibility Metric	Description	Application
f_av,t_	Fraction of API (per dose) bioaccessible (available for absorption) at t h.	F_av,3_ has been identified as the most accurate predictor of relative AUC changes in vivo for fasted state TIM-1 [24]. F_av,5_ indicates the bioaccessible fraction at the end of the experiments in this study.
f_av_,_max_	Maximum amount of API bioaccessible (available for absorption) in a predetermined interval (often referred to as BA_max_ but not to be misinterpreted as maximum bioavailability).	To compare performance of drug products or effects of TIM physiological parameters.
t_max_	Time interval within which f_av_,_max_ occurs.	Used as a proxy for t_max_ in vivo, mostly arising from formulation changes (no PK mechanisms are involved).

## 3. Results and Discussion

The results of the international ring study are presented below, first evaluating reproducibility of total bioaccessibility, followed by analysis of bioaccessibility profiles, intraluminal conditions and TIM bioaccessibility metrics.

### 3.1. Repeatability and Reproducibility of Total Bioaccessible and Recovered Paracetamol

At the end of a TIM experiment, the total bioaccessible amount of paracetamol (f_av_) and recovery (R) following mass balance are key metrics to assess run performance. Table 4 presents a summary of the mean (±standard deviation (SD)) f_av_ (%, at t = 5 h) and recovered paracetamol from experiments in each laboratory, while Table 5 summarizes the intra- and inter-lab variability. Inter-laboratory reproducibility was calculated using laboratory mean values, so that each laboratory contributed equally to the between-laboratory comparison despite differences in replicate number.

For tiny-TIM in the fasted state, the mean f_av_ (%R) across laboratories was 98.5 ± 1.4%, with a narrow inter-laboratory range of 0.9%. In the fed state, f_av_ (%R) averaged 99.2 ± 2.3%, showing slightly greater variability, consistent with the increased complexity of fed-state digestion conditions.

For TIM-1, laboratory 5 conducted the largest number of replicate experiments (n = 6), with f_av_ values of 93.8 ± 5.4%D and 95.4 ± 4.0%D in the fasted and fed states, respectively. When normalized for recovery (f_av_ %R), variability decreased substantially to 87.4 ± 0.9% and 87.4 ± 0.8%, demonstrating good intra-laboratory repeatability. Across laboratories, the mean f_av_ (%R) was 89.5 ± 1.8% in the fasted state and 88.2 ± 1.0% in the fed state, indicating good inter-laboratory reproducibility. Recovery values at laboratory 5 were consistently higher than at the other laboratories. This was in line with slightly higher analytical recovery observed in the QC samples at that site, suggesting that the difference most likely reflects analytical performance rather than a difference in the simulated GI conditions of TIM-1.

Overall, both systems demonstrated good repeatability and reproducibility across laboratories. The bioaccessibility of paracetamol in tiny-TIM was approximately 10% higher than in TIM-1 for all experiments, while recovery of paracetamol from both systems was approximately 100%. As such, the lower bioaccessibility in TIM-1 can be attributed to the presence of an effluent stream that simulates flow from the ileum to the caecum-ascending colon. As a result, a fraction of paracetamol completes transit of the simulated upper GI tract without passing through a filtration unit. In contrast, in this tiny-TIM experiment there was no effluent stream, so dissolved paracetamol was available for filtration over the entire course of the experiment.

In these TIM experiments, the use of an aqueous solution of API rather than a solid oral formulation such as a tablet or capsule eliminated potential variability associated with drug release and dissolution from a solid dosage form. As paracetamol was administered as a solution, hydrodynamic differences between the gastric compartments were not expected to substantially affect bioaccessibility since the same gastric emptying profile was implemented for tiny-TIM and TIM-1. However, such effects may become more relevant when studying solid oral dosage forms, particularly modified release formulations that are more reliant on erosion to achieve release or dosage forms with density-dependent behaviour, such as floating gastro-retentive formulations. Although the unformulated paracetamol might not be fully representative of commercial oral drug products, it allowed for a true evaluation of system performance, independent of any formulation effects.

An additional benefit of paracetamol for this study is its extensive use as a marker of gastric emptying in humans [25]. The results show that the TIM systems can model paracetamol absorption with reproducibility in both fasted- and fed-state conditions. Since the f_2_ analyses were intended to quantify the reproducibility and repeatability of one TIM system, these analyses were restricted to experiments conducted in the same TIM system rather than used as a means to compare tiny-TIM and TIM-1. For further validation of the TIM systems, it would be beneficial to study the inter-laboratory variability in bioaccessibility of poorly soluble compounds (shearing forces, solubilisation of lipophilic compounds, formulation disintegration, etc.); additional and more-challenging APIs and formulations should be investigated.

The number of replicate experiments differed between laboratories (typically n = 2–4, with one laboratory performing n = 6), reflecting the practical constraints of an international ring study. Future interlaboratory studies using additional reference compounds and larger and consistent replicate numbers would further strengthen the generalizability of the validation dataset.

### 3.2. Paracetamol Bioaccessibility Profile

**Figure 2:**
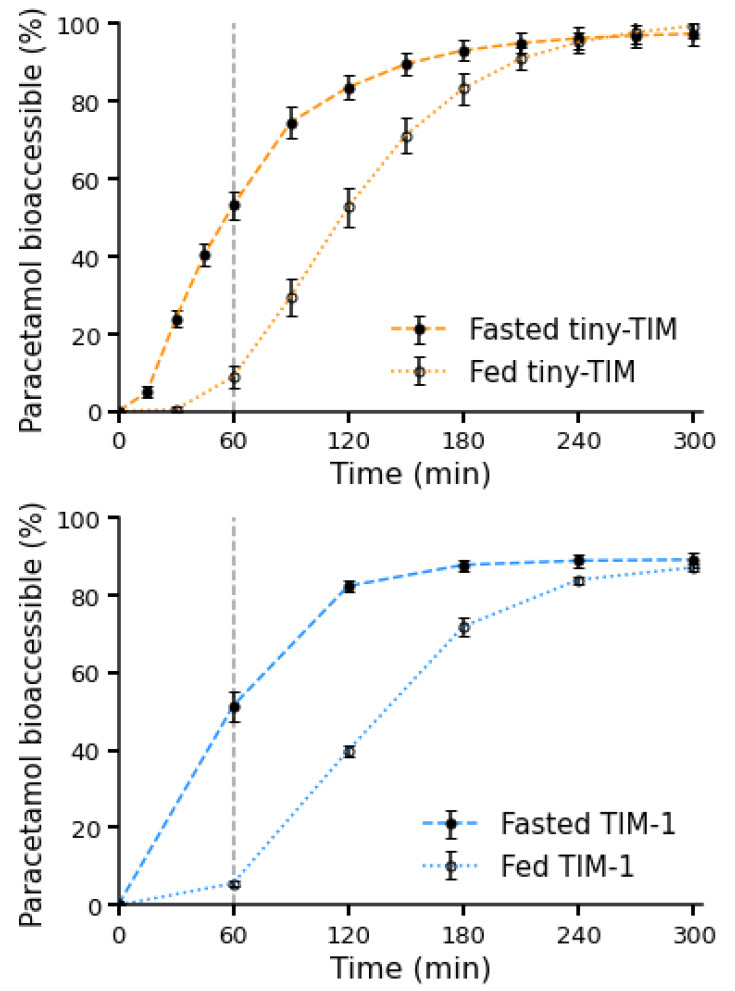
Cumulative bioaccessibility profiles of paracetamol measured in the tiny-TIM and TIM-1. Dashed vertical lines indicate the time of the housekeeper wave (t = 60 min).

Fasted- and fed-state paracetamol bioaccessibility profiles in tiny-TIM and TIM-1 are presented in Figure 2.

The high reproducibility and repeatability of the total bioaccessibility were also evident on a per-time-point basis. At all time points, inter-laboratory results deviated by less than 5%. In TIM-1, variability decreased after the HKW from a maximum of 3.8 to 1.8% (fasted) and 2.3 to 1.0% (fed). This effect was less pronounced for tiny-TIM, with variability ranging between 1.5–4.0% (fasted) and 0.6–5.2% (fed). While there is no universally defined regulatory variability threshold for dynamic non-compendial GI models, variability can be contextualized against established compendial dissolution data quality benchmarks. Regulatory guidance for dissolution profile comparison using f_2_ and USP <1092> considers datasets to be “highly variable” when the relative standard deviation exceeds ~20% at early time points (≤10 min) or ~10% at later time points [26,27]. In this ring study, inter-laboratory variability for total bioaccessibility was <10% and per-time-point variability was ≤5.2% across conditions, indicating high reproducibility for a complex dynamic system.

In both systems, the rate of paracetamol bioaccessibility was slower in the fed state due to delayed gastric emptying; however, early onset of bioaccessible drug was still detected. In TIM systems, while gastric emptying is delayed in the fed state compared to the fasted state, size exclusion by the computer-controlled pyloric valve discriminates the emptying profile of liquids and solids; only particles smaller than approximately 3–5 mm can pass the pyloric valve. When a meal is administered in the fed state, the liquid fraction of the meal empties faster than the solid fraction, as demonstrated in Bellmann et al. [20]. As such, dissolved paracetamol in the gastric compartment may become non-homogenously distributed as some dissolved drug is emptied faster in the liquid fraction of the meal, while the presence of solids delays emptying of liquid containing dissolved drug. This is consistent with in vivo observations of paracetamol emptying from the stomach in the presence of food [28]. Interestingly, Bartholomé et al. found that meals with different caloric densities had different gastric emptying rates but indiscernible paracetamol PK profiles, attributed to food–drug interactions and mixing of the stomach contents. This highlights the value of the TIM systems as part of the biopharmaceutics toolbox, as many other in vitro tools typically do not permit in situ digestion and transit of real food [7] and in silico mechanistic models typically overlook hydrodynamic effects [29].

**Figure 3:**
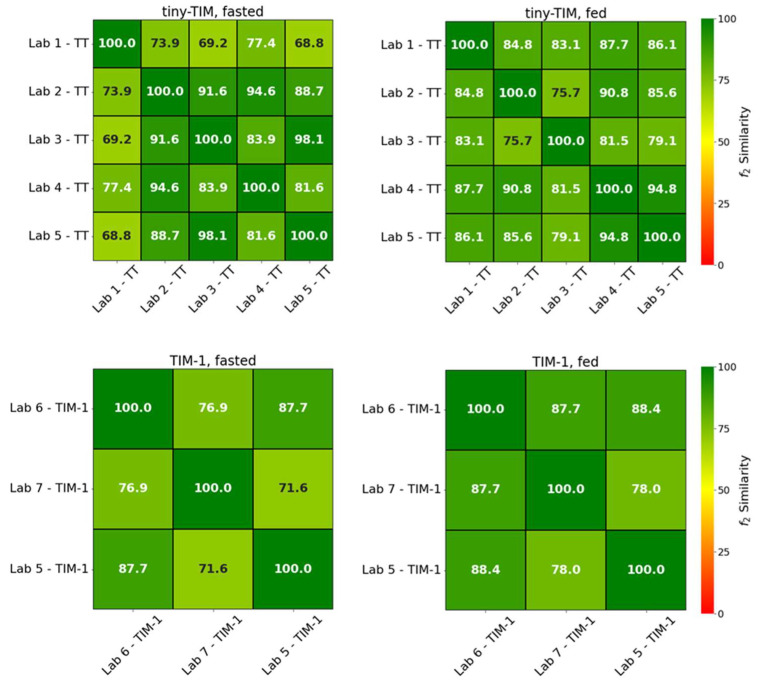
Pairwise f_2_ results of analysis of mean paracetamol bioaccessibility profiles in tiny-TIM (**top**, TT) and TIM-1 (**bottom**) between labs.

Figure 3 summarizes the similarity factor (f_2_) values for each group, along with the number of replicate comparisons. In both the fasted and fed states, the bioaccessibility profile for paracetamol was reproducible for both TIM systems, shown visually by the small error bars (standard deviation) of the mean paracetamol bioaccessibility profiles (Figure 2) and statistically with all f_2_ values > 50 (generally accepted as similar profiles) for comparison of mean paracetamol bioaccessibility profiles between laboratories (Figure 3). The high similarity observed between laboratories therefore supports the robustness of the TIM systems and their application in formulation development.

The f_2_ similarity factor is widely used in regulatory dissolution guidance to compare dissolution profiles of immediate-release drug products. Although TIM systems represent dynamic biorelevant GI models rather than compendial dissolution apparatuses, the f_2_ metric provides a useful approach for comparing cumulative bioaccessibility profiles generated under standardized conditions. It must be emphasized that in the present study, f_2_ was applied as a quantitative measure of repeatability and inter-lab reproducibility rather than as a regulatory dissolution acceptance criterion.

Direct statistical comparison between tiny-TIM and TIM-1 was not performed, as the primary objective of this ring study was to evaluate reproducibility within each system. Comparative evaluations of the two systems have been reported previously [21]. Future studies involving more challenging APIs and complex formulations would further extend validation of the TIM models.

### 3.3. TIM Bioaccessibility Metrics

**Figure 4:**
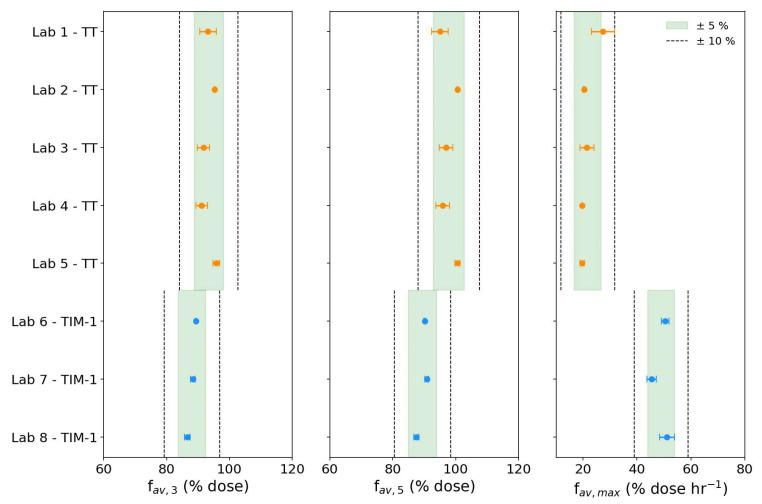
Fasted-state TIM bioaccessibility metrics, presented as geometric mean ± coefficient of variation (CV%) for all replicates per lab for the tiny-TIM (TT, orange data) and TIM-1 (blue data). Horizontal error bars represent the CV% across replicates within each lab. The shaded band indicates ± 5% (green) and the dotted lines bound ± 10% based on the group-level geometric means for tiny-TIM (upper band) and TIM-1 (lower band) to visualize reproducibility.

**Figure 5:**
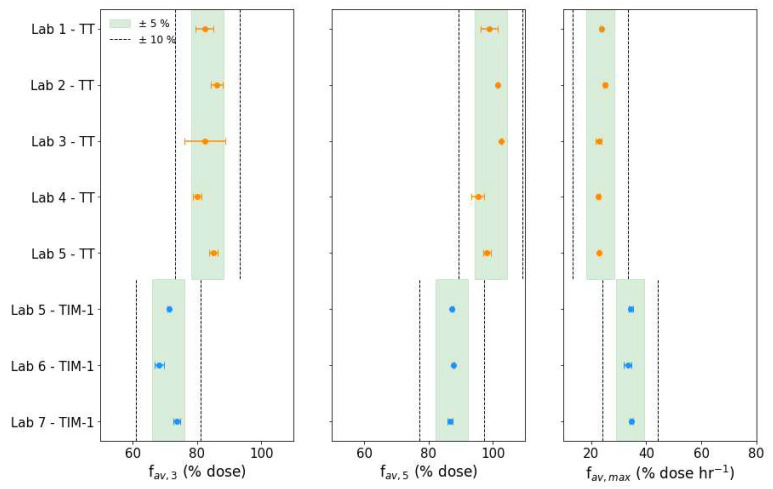
Fed-state TIM bioaccessibility metrics, presented as geometric mean ± coefficient of variation (CV%) for all replicates per lab for the tiny-TIM (TT, orange data) and TIM-1 (blue data). Horizontal error bars represent the CV% across replicates within each lab. The shaded band indicates ± 5% (green) and the dotted lines bound ± 10% based on the group-level geometric means for tiny-TIM (upper band) and TIM-1 (lower band) to visualize reproducibility.

The TIM bioaccessibility metrics were calculated for tiny-TIM and TIM-1 in each laboratory and the results are presented in Figure 4 and Figure 5 in the fasted and fed states, respectively. The figures show the mean f_av,3_, f_av,5_, and f_av_,_max_ (%D) for each lab, with horizontal error bars to indicate the CV (%) across replicates. The shaded regions indicate ±5% and the dashed lines delineate ±10% bounds relative to the group mean (considering tiny-TIM and TIM-1 to be separate groups), providing a visual representation of intra- and inter-laboratory variability.

TIM bioaccessibility metrics may be used to support comparative assessment of drug product performance. While no official regulatory guidance exists for evaluating variability of such measurements derived from biorelevant in vitro models, it is informative to contextualize the observed variability against established benchmarks for in vivo bioequivalence. For example, FDA and EMA guidelines state that for bioequivalence (BE) testing, the 90% confidence interval for PK metrics should fall within 80–125% of the reference product. 

The f_av,3_ describes the bioaccessible fraction of paracetamol at the 3 h mark. In fasted state, f_av,3_ exceeded 80% in both systems and showed low variability, with values within ±5% of their group mean. In the fed state, tiny-TIM f_av,3_ also exceeded 80%, whereas lower values (approximately 70–75%) were observed for TIM-1, with variability within ±10%. Increased variability under fed state conditions is expected due to the added complexity. 

Barker et al. [24] found that the f_av,3_ from a fasted state TIM-1 experiment was most biopredictive of relative changes in fasted state exposure (AUC) in vivo for immediate release (IR) formulations of poorly soluble compounds. This ring study demonstrates that this metric is also reproducible across laboratories for both tiny-TIM and TIM-1, supporting its utility as a performance measure in biopharmaceutics. 

The total fraction bioaccessible at the end of the 5 h experiments, f_av,5_ (summarized previously in Table 4) was >95% in tiny-TIM and >85% in TIM-1. These results indicate that for IR formulations of highly soluble compounds it may be sufficient to run fasted-state experiments in tiny-TIM and TIM-1 for 3 h rather than 5 h, as the extra time may not provide substantial extra information or improvements in terms of variability. 

For the fed state, lower intra-lab variability was observed for f_av,5_ than for f_av,3_, though the inter-lab variability remained similar. Therefore, longer experiments remain appropriate for fed-state conditions, where bioaccessibility is less complete and more variable below 5 h.

Despite the complexity of the systems compared to conventional in vitro dissolution apparatus, both tiny-TIM and TIM-1 experiments produced highly repeatable and reproducible bioaccessibility metrics under simulated fasted and fed state conditions, all of which fell within ±10% of group means.

**Figure 6:**
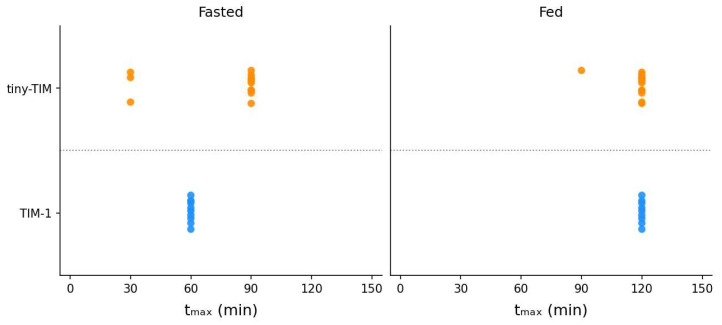
TIM in vitro t_max_ values per replicate in the fasted (left) and fed (right) states. Each dot represents an individual run (orange: tiny-TIM; blue: TIM-1). The dense clustering of points at a single time point within each system shows the high reproducibility of t_max_.

The dense clustering of t_max_ data in Figure 6 with few outliers indicates high reproducibility across laboratories. In the fasted state, tiny-TIM paracetamol t_max_ was 90 min in most experiments (10/13), with three experiments (two from Lab 3, one from Lab 5) showing an earlier t_max_ of 30 min, while TIM-1 consistently showed a t_max_ of 60 min. In the fed state, t_max_ was consistently 120 min for tiny-TIM (in 12/13 replicates) and TIM-1 (in 13/14 replicates), with a single outlier in Lab 5 for each system. It should be noted that the use of 60 min sampling intervals in TIM-1 in this study limited temporal precision and may inflate the apparent magnitude of the fed-state delay in t_max_. Accordingly, TIM-1 t_max_ should be interpreted as a rough descriptor of delayed absorption timing in this study rather than as a precise surrogate for clinical t_max_ under the current protocol.

Typically, in clinical studies, single pharmacokinetic (PK) metrics such as C_max_, t_max_, and AUC are preferred to summarize the pharmacokinetic profile of oral drug formulations and assess drug performance. It must be emphasized that TIM systems are in vitro models of GI transit and in situ dissolution and absorption that lack the full complexity of in vivo absorption, distribution, metabolism, and elimination (ADME) processes that influence these parameters in vivo. While the combination of intraluminal TIM sample-based permeability assays and PBBM/PBPK modelling may overcome these limitations and be a powerful toolbox for complex biopharmaceutics problems, the metrics obtained from TIM experiments alone still serve as useful proxies for estimating corresponding in vivo parameters in in vitro–in vivo-relationship (IVIVR) studies.

On the applicability of TIM bioaccessibility metrics as a proxy to predict in vivo PK, there are two possible avenues to pursue. Firstly, as an IVIVR methodology to directly predict AUC in vivo, which requires specific knowledge of the downstream bioavailability-limiting processes, such as gut wall permeability and first-pass metabolism, as part of a validated PBPK model/PBBM for the compound of interest. The second approach. extensively demonstrated by Barker et al. [24], is to predict relative changes in AUC and C_max_ between formulations. For TIM-1, f_av,3_ was predictive of relative in vivo (humans and dogs) AUC (84%) and C_max_ (79%) for poorly soluble compounds. TIM-1 correctly predicted 84% of rank order cases where a significant food effect or no food effect was observed in humans and dogs, using mostly poorly soluble compounds [18]. This led the authors to state that the TIM-1 can be used as a substitute for dog studies for formulation assessment. More recently, Engman et al. [12] reported that by applying TIM as an advanced dissolution and absorption test in combination with PBBM as a biopharmaceutics bridging risk assessment (BBRA) tool, in vivo bridging studies could be reduced by 70% on average [12]. In this study, standard protocols were designed with testing repeatability and reproducibility in mind, rather than biopredictivity. While it is possible to revise TIM experimental protocols/parameters post-experiment to improve biopredictivity for a specific compound, group of compounds or formulation, this was beyond the scope of the present study.

The filtration mechanism in TIM systems is principally driven by solvent drag across a size-exclusion filter with no cell-mediated effects (permeability, efflux transport, etc.). Since fluid filtration rates vary over time, the concentration profile of a drug in the filtrate alone may not be a strong proxy for the predicted amount absorbed at each time point; hence, f_av_,_max_ was used in this work. Further, comparisons of TIM bioaccessibility metrics between API must be done with caution, since there may be dissimilar retention in the filter due to different affinities for the filter membrane material. In the case of high filter binding, combination of luminal data with in silico modelling may be valuable. However, this limitation does not apply in this work since the same API and filter membrane were used for all experiments.

### 3.4. TIM Luminal Conditions

**Figure 7:**
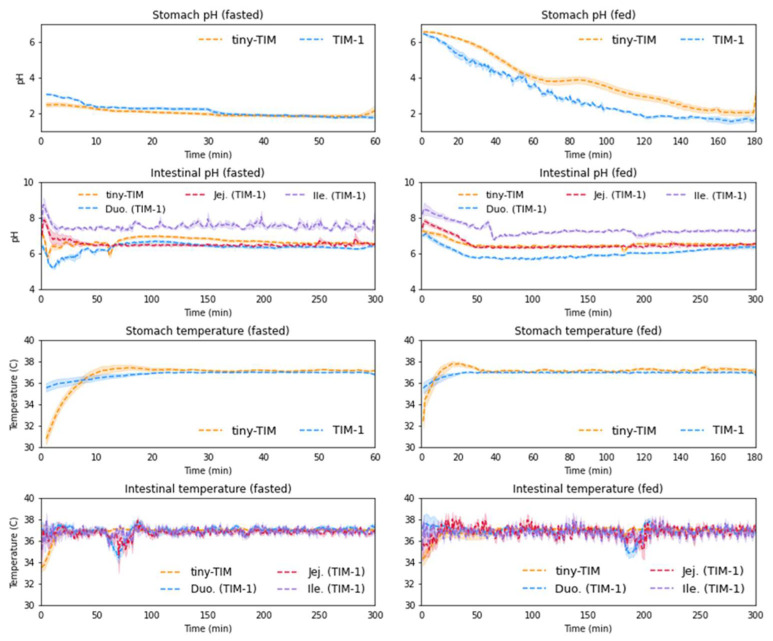
Luminal conditions measured using PAT in the tiny-TIM and TIM-1 (mean data ± standard error of the mean for all laboratories) for the fasted state (left) and the fed state (right). Duo—Duodenum; Jej—jejunum; Ile—ileum.

Process analytical technology (PAT) is built into the TIM systems in the form of in-line pH, temperature and pressure probes, control systems, and automated-data-logging capabilities. The luminal pH and temperature profiles in tiny-TIM and TIM-1 are displayed in Figure 7. Overall, the luminal pH and temperature were within the in vivo range reported in the literature, with fluctuations across transit stages that mirrored reported in vivo patterns [30].

All compartments had a temperature control setpoint of 37 °C. The recorded temperatures of all intestinal compartments were comparable between the different experiments on the different TIM-1 systems. The actual temperatures measured depend on the starting temperature (e.g., temperature of the water and meal intake) and environmental temperature, so small deviations from 37 °C are seen. Following initialization of the systems the stomach temperature began at around 36 °C. When the intake was added, the stomach temperature temporarily dropped to 27 °C, and approximately 20 min was needed to restabilize at 37 °C. In vivo observations have shown that intragastric temperature reaches 37 °C after approximately 20–30 min following ingestion of 400 mL of a warm or cold drink [31]. More recently, using the same volume of water as in this study for the fasted state, Schneider et al. [32] reported a slower equilibration time of approximately 20 min for the intragastric temperature to rise from 22 °C to 36 °C measured with the SmartPill^®^. In this study, the fasted-state intake of 240 mL water was preheated to 37 °C, hence the time required to restabilize at 37 °C, as a small amount of heat is lost during administration to the TIM stomach. Less variability was observed in the jejunal and ileal compartments on TIM-1. The temperature of the duodenal compartment of TIM-1 and small-intestinal compartment of tiny-TIM fluctuated more due to gastric emptying of large volumes from the stomach. The same can be said for the pH of the small intestine (tiny-TIM) and duodenum (TIM-1). In both TIM systems, the intestinal compartments had fixed pH values throughout the experiment (indicated by the green line in the plots). The gastric pH decreased over time, because of the pH-controlled HCl secretion. The observed gastric pH curves in the fasted and fed states were comparable throughout the different experiments performed at the seven sites.

In the fed state, higher variability was observed for temperature in both TIM systems for all compartments. This can be attributed to the inhomogeneous distribution and, therefore, non-uniform contact time of the contents with the heated walls of the TIM lumen, which is likely to be exacerbated in the fed state, where the contents are more viscous compared to the fasted state. Additionally, this may be influenced by the solid and liquid components of the meal having different specific heat capacities, although this may be offset when the contents are pre-heated to 37 °C in accordance with standardized TIM protocols. Similarly, larger pH variability was measured in the fed state, which may be attributed to different buffering effects of the different meal components. Additionally, the TIM system uses the physiological bicarbonate buffer system in the intestinal compartments, which has a low buffering capacity and can be inherently variable.

The gastric pH registered by the tiny-TIM during the experiments was comparable between the laboratories, decreasing from around pH 3.0 to 1.8 over 60 min for the fasted state and from around 6.5 to 1.7 over 180 min for the fed state. Measurements are presented up to 60 min (fasted state) and 180 min (fed state), at which point the housekeeper wave takes place. This empties the stomach, meaning the gastric pH electrode was no longer in contact with GI fluids.

### 3.5. Food Effect on Paracetamol Bioaccessibility in TIM

**Figure 8:**
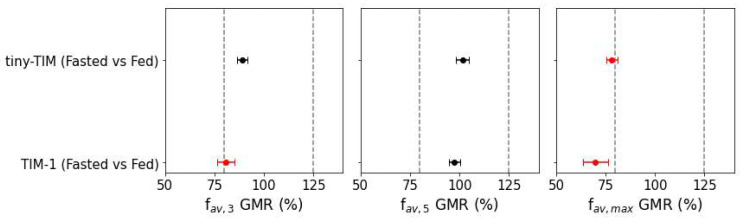
Geometric mean ratios (GMRs) and 90% confidence intervals (CIs) for TIM paracetamol bioaccessibility metrics comparing fasted and fed states. Red data points indicate values where the GMR and corresponding 90% CI fall outside of the 80–125% limits, indicating a food effect.

Figure 8 presents the geometric mean ratios (GMRs) describing the effects of simulated fed state (in vitro “food effect”) on TIM bioaccessibility metrics for 500 mg paracetamol solution. A negative food effect on paracetamol f_av,3_ was observed in tiny-TIM (0.89), which was more pronounced in TIM-1 (0.81). In contrast, f_av,5_ (total fraction bioaccessible) was not significantly affected by fed state in either system (1.01 in tiny-TIM and 0.98 in TIM-1). These findings indicate that the early-stage paracetamol bioaccessibility is more sensitive to fed state conditions in TIM-1 than in tiny-TIM, which reflects differences in intestinal transit and fluid turnover in each system.

A systematic review by Moore et al. [33] concluded that food had no significant effect on paracetamol AUC, but delayed absorption, showing a 32% increase in t_max_. Similarly, no food effect was observed for total paracetamol bioaccessibility (f_av,5_) in either TIM system, while a 33% increase in t_max_ was observed in tiny-TIM (Figure 6). In TIM-1, a twofold increase in t_max_ was measured. This delay in bioaccessibility is consistent with the system parameters governing slower gastric emptying under simulated fed-state conditions. However, t_max_ measurements are inherently influenced by the limited temporal resolution of the data of both systems, which was more pronounced in TIM-1 (60 min). In TIM-1, this may be further compounded by the absence of a duodenal filtration unit, as highlighted in Sarcevica et al. [34]. These findings indicate that food effect on paracetamol t_max_ in tiny-TIM was biopredictive of the food effect in vivo (human), while the absence of a food effect on total bioaccessibility in both systems was consistent with the lack of food effect on paracetamol AUC in humans. Future work should sample with a higher temporal resolution in TIM-1 if the aim is to generate biopredictive data, particularly concerning the estimation of t_max_. Previous work successfully generated a predictive level A IVIVC for paracetamol using TIM-1, where USP II methods failed to do so [13]. In that study, TIM-1 filtrate sampling intervals had a higher resolution than those of the present study. However, a time-shift in the bioaccessibility data was required given the rapid in vivo absorption of paracetamol solution. This limitation could instead be addressed by incorporating intraluminal data to the biopharmaceutics modelling approach or applying a higher filtration rate during the TIM experiment [6].

In both tiny-TIM and TIM-1, f_av_,_max_ was significantly reduced in the fed state to 78 and 71% in tiny-TIM and TIM-1 respectively, due to the delayed gastric emptying and more evenly distributed filtration over the duration of the experiment. In vivo, paracetamol plasma C_max_ was found to be reduced in the fed state to 58% on average of the fasted state value. Despite TIM results (f_av_,_max_.) capturing the direction of food effect on C_max_, there is room for improvement regarding the magnitude.

## 4. Conclusions

This international ring study demonstrated that the tiny-TIM and TIM-1 in vitro models reproducibly simulate gastrointestinal conditions across laboratories, despite the complexity of the dynamic mass transfer processes involved, including gastric emptying, peristaltic mixing, secretion, digestion and intestinal filtration. This consistent simulation of physiological conditions enabled robust and reproducible measurements of paracetamol bioaccessibility under both fasted and fed state conditions with variability within established regulatory benchmarks for dissolution data quality. Additionally, the TIM systems generated repeatable single-point metrics that may be used as a proxy for predicting relative product performance and food effects in vivo. As the most extensive inter-laboratory validation study of the TIM systems to date, these data support their use as biorelevant tools in biopharmaceutics applications and provide a foundation for future regulatory interactions involving TIM data. 

## Figures and Tables

**Table 1:** Participating laboratories.

Lab #	Affiliation	Location	Tiny-TIM	TIM-1
1	Abbvie	Ludwigshafen, Germany	✓	
2	Johnson & Johnson	Beerse, Belgium	✓	
3	AstraZeneca	Macclesfield, UK	✓	
4	Boehringher Ingelheim Pharmaceuticals, Inc.	Ridgefield, CT, USA	✓	
5	InnoGI Technologies	Delft, The Netherlands	✓	✓
6	PozLab *	Poznan, Poland		✓
7	Pfizer Inc.	Sandwich, UK		✓

* PozLab (Poznan) performed the experiments on behalf of GlaxoSmithKline Pharmaceuticals.

**Table 2:** Overview of system parameters, corresponding control mechanisms and fasted- and fed-state setpoints in tiny-TIM and TIM-1. The gastric pH control endpoint was at t = 60 and 180 min in simulated fasted and fed states respectively. GE: Gastric emptying. HKW: Housekeeper wave time point. t_50_ is the time taken (min) to empty half of the contents from the stomach and β (dimensionless) controls the emptying rate.

System	Parameter	Control	Fasted Setpoint	Fed Setpoint
All	GE (HKW (min), t_50_ (min), β)	Level sensor	60, 20, 1.0	180, 80, 2.0
	Gastric pH (start)	HCl secretion	3.0 ± 0.2	6.5 ± 0.2
	Gastric pH (end)		1.7 ± 0.2	1.7 ± 0.2
TIM-1	Duodenal pH	Bicarbonate secretion	6.3 ± 0.2	5.9 ± 0.2
	Jejunal pH		6.5 ± 0.2	6.5 ± 0.2
	Ileal pH		7.4 ± 0.2	7.4 ± 0.2
tiny-TIM	Small-intestinal pH		6.5 ± 0.2	6.5 ± 0.2

**Table 4:** Results overview of tiny-TIM and TIM-1 experiments performed by the seven international laboratories. The recovery (R) of paracetamol as a percentage of the dose and the total fraction bioaccessible (available for absorption, f_av,5_, at t = 5 h) as a percentage of dose and recovery (%D and %R, respectively). Total indicates the mean of the lab mean values.

	Laboratory							
	# 1	# 2	# 3	# 4	# 5	# 6	# 7	Total
	tiny-TIM fasted state							
	n = 3	n = 2	n = 4	n = 2	n = 2			
R (%)	97.5 ± 3.1	102.1 ± 1.0	99.1 ± 2.1	97.0 ± 2.4	101.9 ± 0.9			99.52 ± 2.3
f_av,5_ (%R)	98.4 ± 1.3	98.6 ± 0.6	98.0 ± 2.8	98.9 ± 0.2	98.8 ± 0.1			98.54 ± 1.4
f_av,5_ (%D)	95.0 ± 3.1	100.6 ± 0.4	96.1 ± 2.1	95.9 ± 3.1	100.6 ± 1.2			97.64 ± 2.5
	tiny-TIM fed state							
	n = 3	n = 2	n = 3	n = 2	n = 3			
R (%)	101.8 ± 3.4	103.1 ± 0.0	105.9 ± 1.3	97.2 ± 2.4	99.3 ± 1.2			101.46 ± 3.4
f_av,5_ (%R)	97.2 ± 0.2	98.6 ± 0.4	103.1 ± 1.4	98.1 ± 0.6	98.9 ± 0.6			99.2 ± 2.3
f_av,5_ (%D)	98.9 ± 3.3	101.5 ± 0.5	102.7 ± 0.2	95.4 ± 2.8	98.2 ± 1.6			99.34 ± 2.8
	TIM-1 fasted state							
					n = 6	n = 2	n = 2	
R (%)					107.3 ± 5.2	99.6 ± 1.1	94.0 ± 1.0	100.3 ± 6.7
f_av,5_ (%R)					87.4 ± 0.9	90.2 ± 0.3	90.8 ± 0.9	89.5 ± 1.8
f_av,5_ (%D)					93.8 ± 5.4	89.7 ± 1.3	85.3 ± 1.8	89.6 ± 4.3
	TIM-1 fed state							
					n = 6	n = 2	n = 2	
R (%)					109.2 ± 4.9	97.1 ± 0.5	96.7 ± 0.8	101.0 ± 7.0
f_av,5_ (%R)					87.4 ± 0.8	88.0 ± 0.6	89.3 ± 0.4	88.2 ± 1.0
f_av,5_ (%D)					95.4 ± 4.0	85.4 ± 1.1	86.3 ± 0.3	89.0 ± 5.4

**Table 5:** Summary of intra- and inter-laboratory variability for paracetamol bioaccessibility (f_av_, %R) in tiny-TIM and TIM-1. Intra-laboratory repeatability is represented by the standard deviation of the laboratory with the largest number of replicate experiments; where multiple laboratories performed the same number of replicates, the highest SD was selected. Inter-laboratory reproducibility is expressed as the standard deviation of laboratory mean values. The inter-laboratory range is defined as the difference between the maximum and minimum laboratory mean values.

		Repeatability	Reproducibility	
System	State	Intra-Lab SD (%R)	Inter-Lab SD (%R)	Inter-Lab Range (%R)
tiny-TIM	Fasted	2.8 (Lab 3, n = 4 experiments)	0.4 (n = 5 labs)	0.9
	Fed	1.4 (Lab 3, n = 3 experiments)	2.3 (n = 5 labs)	5.9
TIM-1	Fasted	0.9 (Lab 5, n = 6 experiments)	1.8 (n = 3 labs)	3.4
	Fed	0.8 (Lab 5, n = 6 experiments)	1.0 (n = 3 labs)	1.9

## Data Availability

The original contributions presented in this study are included in the article. Further inquiries can be directed to the corresponding author.

## Appendix A. Geometric Mean Calculations

To evaluate bioaccessibility metrics between groups (TIM system or fasted and fed states), GMRs were calculated. To facilitate this, the mean difference (MD) between group A and B (for example) was first computed (in log space):

(A1) $$MD=\log\left({GM}_{A}\right)-\log\left({GM}_{B}\right),$$

where $GM_{A}$ and $GM_{B}$ are the geometric means of groups A and B, respectively. Using replicate-level log-transformed data, the standard error (SE) was calculated:

(A2) $$SE\;=\;\frac{\sigma }{\sqrt{n}}$$

where σ is standard deviation (of the log-transformed data in this case) and n is the number of replicates. Degrees of freedom (df) were estimated using the Welch–Satterthwaite equation:

(A3) $$df=\;\frac{{\left({SE}_{A}^{2}+{SE}_{B}^{2}\right)}^{2}}{\frac{{SE}_{A}^{4}}{n_{A}-1}+\frac{{SE}_{B}^{4}}{n_{B}-1}}$$

where nA and nB are the number of replicates in groups A and B respectively and $SE_{A}$ and $SE_{B}$ are the standard errors of groups A and B. The Standard Error of the Difference (SED) between groups was calculated next:

(A4) $$SED\;=\;\sqrt{{SE}_{A}^{2}+\;SE_{B}^{2}}$$

And the GMR was then obtained with Equation (A5), where it is expressed as a percentage:

(A5) $$GMR=\exp\left(MD\right)\times 100$$

Finally, the confidence interval was calculated with Equation (A6):

(A6) $$CI=e^{\left(\mathrm{MD}\pm t_{\alpha /2,\mathrm{df}}\cdot \mathrm{SED}\right)}\times 100$$

where $t_{\alpha /2,df}$ is the critical value from the Student’s t-distribution at significance level α (two-sided), df is degrees of freedom and α = 0.10 for 90% confidence intervals.
